# Efficacy of navigation may be influenced by retrosplenial cortex-mediated learning of landmark stability

**DOI:** 10.1016/j.neuropsychologia.2017.08.012

**Published:** 2017-09

**Authors:** Stephen D. Auger, Peter Zeidman, Eleanor A. Maguire

**Affiliations:** Wellcome Trust Centre for Neuroimaging, Institute of Neurology, University College London, 12 Queen Square, London WC1N 3BG, UK

**Keywords:** Retrosplenial, Navigation, FMRI, Landmarks, Permanence, Human, Virtual reality

## Abstract

Human beings differ considerably in their ability to orient and navigate within the environment, but it has been difficult to determine specific causes of these individual differences. Permanent, stable landmarks are thought to be crucial for building a mental representation of an environment. Poor, compared to good, navigators have been shown to have difficulty identifying permanent landmarks, with a concomitant reduction in functional MRI (fMRI) activity in the retrosplenial cortex. However, a clear association between navigation ability and the learning of permanent landmarks has not been established. Here we tested for such a link. We had participants learn a virtual reality environment by repeatedly moving through it during fMRI scanning. The environment contained landmarks of which participants had no prior experience, some of which remained fixed in their locations while others changed position each time they were seen. After the fMRI learning phase, we divided participants into good and poor navigators based on their ability to find their way in the environment. The groups were closely matched on a range of cognitive and structural brain measures. Examination of the learning phase during scanning revealed that, while good and poor navigators learned to recognise the environment's landmarks at a similar rate, poor navigators were impaired at registering whether landmarks were stable or transient, and this was associated with reduced engagement of the retrosplenial cortex. Moreover, a mediation analysis showed that there was a significant effect of landmark permanence learning on navigation performance mediated through retrosplenial cortex activity. We conclude that a diminished ability to process landmark permanence may be a contributory factor to sub-optimal navigation, and could be related to the level of retrosplenial cortex engagement.

## Highlights

• People learned the layout of a virtual environment during fMRI scanning.
• Wayfinding was tested after the learning phase and good/poor navigators identified.
• Poor navigators were impaired at registering landmark permanence during learning.
• This was accompanied by reduced retrosplenial cortex activity.

## 1 Introduction

Behavioural and brain differences between good and poor navigators have been widely reported (Auger et al., 2012, Auger and Maguire, 2013, Baumann et al., 2010, Epstein et al., 2005, Hartley et al., 2003, Janzen et al., 2008, Maguire et al., 2000, Ohnishi et al., 2006, Sulpizio et al., 2016, Wegman and Janzen, 2011, Woollett and Maguire, 2011), but the specific causes of navigation variability have been more difficult to determine (Wolbers and Hegarty, 2010). Effective navigation relies upon the formation and utilisation of accurate environmental representations, the bedrock of which are stable landmarks (Burnett et al., 2001, Lynch, 1960, Siegel and White, 1975). These landmarks can be distal, global cues (Doeller et al., 2008) or more proximal objects (Committeri et al., 2004, Galati et al., 2010, Lew, 2011, Marchette et al., 2015, Marchette et al., 2014, Yoder et al., 2011), but whatever the size or salience of these permanent, non-moving environmental features, how they are processed by the brain may be related to a person's general navigation ability (Auger et al., 2012, Auger and Maguire, 2013).

A previous functional MRI (fMRI) study demonstrated that the retrosplenial cortex (RSC) was responsive to the permanence of common everyday landmarks (Auger et al., 2012). Moreover, processing of permanence appeared to be automatic, being implicitly registered even when attention was not directly drawn to this landmark feature. Interestingly, relative to good navigators, poor navigators had a specific deficit in reliably identifying the most permanent, non-moving items in the environment, and reduced responses to permanent landmarks in the RSC (Auger et al., 2012). It has also been shown that RSC codes for the specific number of permanent items in view, and the RSC of good navigators contained more discriminative representations of these permanent landmarks (Auger and Maguire, 2013). Other work has revealed that representations of permanence in RSC developed rapidly for completely novel items, and RSC responses directly tracked the emerging knowledge of landmark permanence (Auger et al., 2015).

Processing of other landmark features, such as whether or not items are encountered at navigationally relevant ‘decision points’ in an environment (Janzen and van Turennout, 2004, Schinazi and Epstein, 2010), whether they evoke a sense of surrounding space (Mullally and Maguire, 2011), their size and visual salience (Auger et al., 2012, Konkle and Oliva, 2012), have been found to engage other brain regions, in particular the parahippocampal cortex (PHC). Responses in PHC have also been linked to general navigation abilities (Wegman and Janzen, 2011).

The hippocampus (HC) is the other brain region where there is extensive evidence for a role in navigation ability (Bohbot et al., 2007, Hartley et al., 2003, Iaria et al., 2008, Janzen et al., 2008, Maguire et al., 2000, Schinazi et al., 2013, Wegman and Janzen, 2011, Woollett and Maguire, 2011). Unlike RSC and PHC, however, the HC has not been found to operate at the basic level of individual landmark features. Instead, the HC appears to be associated with the processing of more detailed spatial information related to knowledge of where landmarks are situated in an environment overall (Auger et al., 2015), consistent with its often reported role in retrieving spatial location information about objects (Baumann et al., 2010, Ekstrom et al., 2011, Manns and Eichenbaum, 2009, Save et al., 1992).

Thus, there are numerous examples of MRI studies linking RSC, PHC and HC with navigation ability, and also with landmark features, in particular permanence. However, no study has directly examined the relationship between good and poor navigation and the learning of landmark permanence, along with the concomitant fMRI activity. To address this issue, we first needed to identify groups of good and poor navigators by objectively measuring their wayfinding in an environment that they had all learned, and then somehow retrospectively assess how they had come to learn about the permanence of landmarks within that environment, all while in an MRI scanner. It was also important that the landmarks in question were novel, so that participants did not have prior knowledge or expectations about their permanence status, but had to acquire this knowledge when learning the environment.

**Fig. 1:**
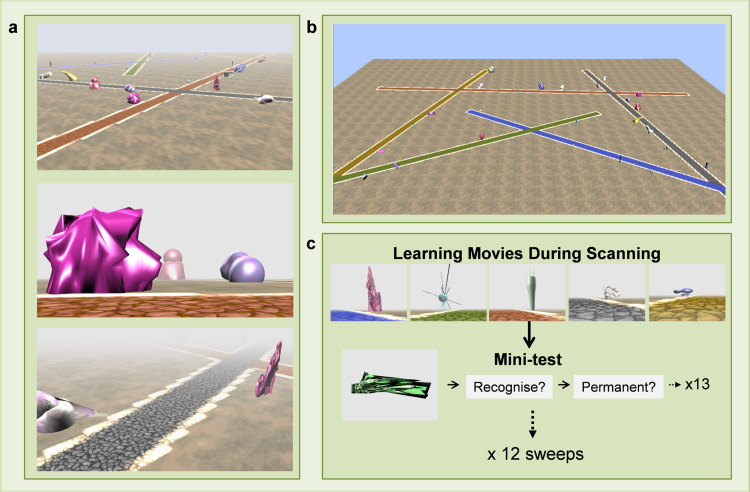
*The virtual reality environment and fMRI task.* (a) Screenshots showing landmarks situated alongside the 5 different coloured paths. Fog was used to control subjects’ exposure to the environment. (b) An aerial perspective without fog showing how the 5 paths related to one another. (c) The learning phase during fMRI consisted of 12 learning “sweeps”.

We therefore created a virtual reality environment containing five overlapping paths and landmarks of which participants had no prior experience (Fig. 1). Participants learned the layout of this environment while undergoing fMRI scanning knowing that their knowledge of the environment would be tested in a variety of unspecified ways after scanning. Crucially, of the environment's 60 landmarks, some remained fixed in their locations while others changed position each time they were seen (Auger et al., 2015). Participants’ knowledge of landmark identity (recognition memory) and permanence was assessed during and after the fMRI learning scan. Also after scanning, we examined how well they knew the overall layout of the environment and, importantly, their ability to navigate within it.

We reasoned that the most obvious and naturalistic way to divide participants into good and poor navigator groups was based on their ability to find their way within the environment after the learning phase in the scanner. We could then look back at both the learning and fMRI data that were acquired during scanning to examine whether there were any differences between good and poor navigators. Given previous reports (Auger et al., 2012, Auger and Maguire, 2013), we predicted that poor, relative to good, navigators would be significantly worse at learning landmark permanence. We also expected that this would be accompanied by reduced activity specifically in the RSC of poor navigators during learning.

## 2 Materials and methods

### 2.1 Participants

Thirty two subjects (16 female, mean age 23.7 years, SD 2.4) took part in the experiment. All were right handed and healthy with normal vision. The participants and experimental design have been reported previously (Auger et al., 2015) in a study that was focused on a different set of questions which did not involve the data presented here. All experimental protocols were approved by the University College London Research Ethics Committee. The experimental methods were carried out in accordance with the approval of the Ethics Committee. Informed written consent was obtained from all participants.

### 2.2 The virtual environment

The virtual environment has been described elsewhere (Auger et al., 2015); details are reprised here for convenience. It was created using the jMonkeyEngine 3.0 beta game engine (http://jmonkeyengine.org), Java JDK 1.6 (Sun Microsystems, Santa Clara, California) and Blender (Stichting Blender Foundation, Amsterdam). The world contained 5 different coloured intersecting straight paths (yellow, red, grey, blue and green). The landmarks were developed and characterised in a previously-published study involving separate participants (Auger et al., 2015). This ensured that the permanent and transient landmarks were matched in terms of visual salience, how well they could be remembered, as well as other features. Each path had 12 landmarks (6 permanent, 6 transient) evenly distributed alongside it (Fig. 1a, b). A trial consisted of travelling along one of these paths. There were 60 trials in total, with the 5 paths being travelled 12 times each. Permanent landmarks remained in the same location on each trial, whereas transient landmarks appeared in a different location on every exposure. The locations in which all 60 landmarks appeared on each of the 60 trials were meticulously designed so that permanent and transient landmarks were equally distributed either side and along the whole length of each path. This ensured that the permanent and transient landmarks, as well as being matched for their perceptual features (size, visual salience, and other features – see Auger et al., 2015), were placed in equivalent locations within the environment.

To precisely control learning, participants were exposed to the environment by viewing first person perspective movies travelling along each of the 5 paths, one at a time. Each movie took a first person perspective travelling along one of the paths. In these movies, the environment was covered in a shroud of fog to restrict the field of view and ensure close control over participants’ exposure to the landmarks, hence we refer to the environment as ‘Fog World’.

On each trial, the camera travelled along a path in a straight line. When a landmark emerged out of the fog, the camera turned to bring the landmark into the centre of the screen, where it was positioned for 2 s, the camera then panned back to the middle of the path as it continued travelling forwards. The paths were always travelled in the same direction, with the same start and end point each time. Each trial consisted of a single journey along one of the paths and at the end of a movie subjects were immediately shown the next learning trial on a different path. During scanning, the ordering of trials along the 5 different paths was pseudorandomised.

To encourage subjects to learn an integrated representation of the whole environment, the paths intersected one another. Each path intersected with two others. The first intersection was located 3 landmarks after the start of the path and the second was 3 landmarks before the end, with 6 landmarks between the two intersections. When the movies came to one of these intersections, the camera turned either left or right and the fog cleared sufficiently to reveal 3 landmarks on the adjoining path. After 3 s, the landmarks were obscured by the fog again with the camera returning to the centre while continuing along the route. There were equal numbers of left and right turns at each intersection throughout the whole experiment and the ordering of the turns was pseudorandomised to ensure it was not predictable. The number of times each landmark was viewed during one of these intersection turns was also controlled so that overall exposure to all the landmarks remained identical. Each path movie was approximately 1 min in length.

### 2.3 Tasks

Before scanning, subjects were instructed to learn the layout of the environment and were told they would be tested in a variety of ways after scanning without the specific nature of those tasks being revealed. They were explicitly informed that some of the landmarks would always remain in the same locations (permanent landmarks, n=30) whereas others would appear in a different place every time they were seen (transient landmarks, n=30). They were shown an example trial (containing landmarks and a path which did not appear during the main experiment) to familiarise them with the general format of the main fMRI task. Prior to commencing the experiment, participants also completed the Santa Barbara Sense of Direction (SBSOD) questionnaire which elicits self-reports of typical navigation performance, and is commonly used as a proxy for real-world navigation ability (Hegarty et al., 2002).

During scanning, participants were shown movies travelling along each of the five paths. When all five paths had been travelled once, there was a questioning period to gauge how much information had been learned by that point in the experiment. Participants were first shown an image of a single landmark displayed, in isolation, on a plain grey background for 2 s. They were then asked whether or not they remembered the landmark from the environment (“*Have you seen this item in the environment?*”, Yes/No). If they remembered seeing it, they were then asked about its permanence (“*How many locations in the environment have you seen it in?*”, Only 1/More than 1), before being questioned about another landmark. Within each questioning period, subjects were asked about 13 landmarks: 5 permanent, 5 transient and 3 previously unseen. Each sequence of viewing movies of the 5 paths followed by a questioning period is referred to as a learning ‘sweep’ (Fig. 1c). There were 12 learning sweeps in total.

In the debriefing session after scanning, several questions relating to the landmarks were asked, with a different randomised order of landmarks used for each question. Questions were asked in the following order for each subject: recognition memory, permanence knowledge, ratings of landmark salience, and ratings of landmark size. For the recognition memory test, subjects were shown images of individual landmarks one at a time (all 60 from the environment and 26 novel landmarks) and were asked to decide whether or not they recognised them from the environment (“*Do you remember seeing this item in the environment?*”, Yes/No). After that, questions were only asked about the landmarks from the environment. Participants had to decide whether a landmark was permanent or transient (“*How many positions in the environment do you think this item was in?*”, Only 1/Many). Next they rated the salience (“*To what extent does this item grab your attention*”, Not at all/A bit/A lot) and then the size (“*What size is this item*”, Small/Medium/Large) of each landmark.

Subjects then had three further tests, one of which – drawing a sketch map of the environment –examined overall knowledge of the paths and how they were related, irrespective of landmarks. The second – placing landmarks on a map which featured just the paths – tested knowledge of landmark locations without the need to retrieve knowledge of the paths. The final test – active first-person navigation within Fog World – encompassed all aspects of the environment including paths, their relationships, landmark types and their locations, and was the task that was most like real-life navigation.

#### 2.3.1 Sketch map task

Subjects were handed a blank sheet of A3 paper and a black pen; they were instructed to draw a map of the environment “in as much detail as possible”. They were told to include as much as they could remember about the environment's layout, all the paths and, if possible, the positions of landmarks. The sketch map drawings were later scored according to how accurately the relationships between paths were drawn (ignoring any landmarks that were included), which indicated how well subjects had learned the overall structure of the environment. One point was awarded for each correctly drawn intersection between two paths (e.g., if it included an intersection between red and yellow paths), a further point was awarded if the parts of the paths involved in that intersection were accurate (e.g., if the start of the red path intersected with the end of the yellow path). Thus, a maximum of 2 points were available for each of the environment's five path intersections, giving an overall maximum possible score of 10. Any incorrectly drawn intersections (e.g., the yellow path intersecting with the grey path) were penalised 1 point, with a minimum possible score of 0.

#### 2.3.2 Landmark placement task

After finishing their sketch map, participants were handed a map (on A3 paper) showing the real layout of the environment from an aerial perspective and its five different coloured paths (this was the first time an aerial perspective of the environment had been shown to subjects). However, this map contained no landmarks. They were then shown images of 25 landmarks (21 permanent and 4 transient), one at a time, on a computer screen. Each landmark image had a number next to it and participants were instructed to write that number on the map where they believed the landmark was located. They were told to be as accurate as possible in placing each landmark, being careful which path and which part/side of the path they located it. The map had an additional box in the corner of the page where they could indicate if they thought a landmark was transient. Three points were available for each of the 25 trials: 1 point for placing a landmark next to the correct path (e.g., next to blue path), 1 point for placing it on the correct part of that path (i.e., before the 1st intersection, between the 2 intersections, or after the 2nd intersection) and a final point for placing a landmark on the correct side of the path (right or left). A correctly identified transient landmark was awarded 3 points. The maximum possible score was 75.

#### 2.3.3 Active navigation task

The final active navigation task was performed on a computer. Participants were first shown an image of a landmark and instructed that they would have to navigate to where they thought it was located in the environment by as direct a route as possible. On each trial, subjects were placed within a version of the environment in which there was no fog and the target landmark had been removed. They moved to where they thought that landmark belonged (using the arrow keys on a keyboard) and then indicated their chosen location by pressing the space bar. There were 12 trials in total (9 permanent and 3 transient landmarks). If they thought the target landmark was transient (and so could not be placed in a single location), subjects were instructed to press the space bar and indicate that they thought it was transient. The start point for each of the navigation trials was a large distance away from the target landmark location, requiring participants to correctly traverse multiple intersections between the paths. On each trial a point was awarded only if participants took the most direct route to the correct target location. For transient landmark trials, a point was only awarded if participants immediately recognised that attempting to navigate was futile (by pressing the space bar before any meaningful attempt to navigate). The maximum score on this test was therefore 12 points.

Finally, subjects answered some general questions in a post-scan questionnaire – how difficult they found the task overall (1/very easy…5/very hard), how difficult they found learning the environment (1/very easy…5/very hard) and how much previous experience they had playing video games (1/none…5/very experienced).

### 2.4 Scanning parameters, preprocessing and analyses

T2*-weighted single-shot echo-planar images with blood oxygenation level-dependent (BOLD) contrast were acquired on a 3 T Magnetom Allegra head-only MRI scanner (Siemens Healthcare, Erlangen, Germany) operated with the standard transmit-receive head coil. Functional MRI data were acquired across four sessions each lasting approximately 18 min (allowing participants a short break in between sessions to ensure they could stay engaged during the task). The sequence used was optimised to minimise signal dropout in the medial temporal lobe and used a descending slice acquisition order with a slice thickness of 2 mm, an interslice gap of 1 mm, and an in-plane resolution of 3 × 3 mm (Weiskopf et al., 2006). Forty eight slices angled at −45° to the anterior-posterior axis were collected covering the entire brain, with a repetition time of 2.88 s, 30 ms echo time and 90° flip angle. A 3D MDEFT T1-weighted structural scan was also acquired for each participant with 1 mm isotropic resolution (Deichmann et al., 2004). The first 6 ‘dummy’ volumes from each of the four sessions were discarded to allow for T1 equilibration effects. The total number of volumes acquired in each scanning session was variable due to the variability in length of the inter-sweep questioning periods (e.g., a participant would not answer the permanence question if they said they did not recognise an item). FMRI data were analysed using SPM8 (www.fil.ion.ucl.ac.uk/spm). Images were realigned and unwarped using field maps which were acquired with a double-echo gradient field map sequence (TE=10 and 12.46 ms, TR = 1020 ms, matrix size 64 × 64, with 64 slices, voxel size = 3×3×3 mm) and then normalised to a standard EPI template in MNI space with a resampled voxel size of 3×3×3 mm and smoothed using an 8 mm FWHM Gaussian kernel.

#### 2.4.1 MRI regions of interest (ROIs)

We focused on three regions of interest (ROI): retrosplenial cortex (RSC) and posterior parahippocampal cortex (PHC), due to their previously demonstrated roles in processing features of real-world landmarks in association with navigation ability (Auger et al., 2012, Auger and Maguire, 2013, Janzen et al., 2008, Mullally and Maguire, 2011, Troiani et al., 2014), and the hippocampus (HC), which has been associated with navigation expertise (Bohbot et al., 2007, Hartley et al., 2003, Iaria et al., 2008, Janzen et al., 2008, Maguire et al., 2000, Schinazi et al., 2013, Woollett and Maguire, 2011). The ROIs were defined anatomically, with bilateral masks for each area. These anatomical masks were delineated by an experienced researcher, not involved in this project, on an averaged structural brain scan from a different group of n=30 participants, guided by Duvernoy (1999) and Vann et al. (2009). The RSC mask was delineated according to the Duvernoy atlas’ definition of Brodmann Areas 29 and 30. The PHC mask was anatomically defined to include the posterior portion of the parahippocampal gyrus, distinct from the perirhinal and entorhinal cortices and with the posterior aspect of the parahippocampal gyrus as the posterior boundary. This posterior portion of the parahippocampal gyrus aligned with the functionally defined “parahippocampal place area” (PPA) when compared to the results of a search for “PPA” using the online neurosynth meta-analysis tool (http://neurosynth.org).

#### 2.4.2 Structural MRI analysis

Voxel-based morphometry (VBM; Ashburner and Friston, 2000) analyses were performed that directly compared the whole brain structural MRI scans of the good and poor navigators using a two sample *t*-test with a smoothing kernel of 8 mm full width at half maximum at a whole brain FWE corrected threshold of p<0.05. Comparisons were also made within each of the predefined ROIs, as detailed in the Results section.

#### 2.4.3 Functional MRI analysis

Separate subject-specific regressors were created for the times permanent and transient landmark images were in view during the question periods of each learning sweep (i.e., one regressor for all permanent landmarks and a second for all transient landmarks). These regressors of interest were convolved with the canonical HRF. Additional regressors were created to account for the remaining time period and subject-specific movement during the scan, all of which were treated as covariates of no interest. The first and second halves of the learning phase (learning sweeps 1–6 and 7–12 respectively) were analysed separately in order to examine changes occurring early and later in learning. For each voxel, subject-specific parameter estimates pertaining to each regressor of interest (betas) were calculated. The primary contrast of interest was a direct comparison between the permanent and transient landmark betas. Each subject's contrast map was summarised within each ROI by extracting the first principal component (eigenvariate) using the MarsBar toolbox. The mean of each timecourse was then calculated and used to compare subjects’ responses.

#### 2.4.4 Mediation analysis

To further explore the relationships between navigation performance, permanence learning and fMRI activity within the three ROIs, we conducted mediation analyses (Preacher and Hayes, 2008). A mediation analysis assesses whether a predictor variable (in this instance permanence learning) has a causal effect upon another variable (navigation) via a third, mediator variable (activity in RSC, HC or PHC). Specifically, we considered the relationship between individual subjects’ differences in permanence learning (in the second learning segment during scanning) and later navigation scores (post-scan). We were especially interested in whether or not there was evidence of activity in RSC, HC or PHC in the intervening period (in the second half of learning during scanning) mediating this relationship. In other words, could the differences in fMRI activity demonstrated in Fig. 4 account for some of the behavioural differences demonstrated in Fig. 3 and if so, how much. Mediation analyses were run with the PROCESS macro in SPSS (www.processmacro.org) using 10,000 bootstrap samples.

## 3 Results

### 3.1 Identifying good and poor navigators

**Fig. 2:**
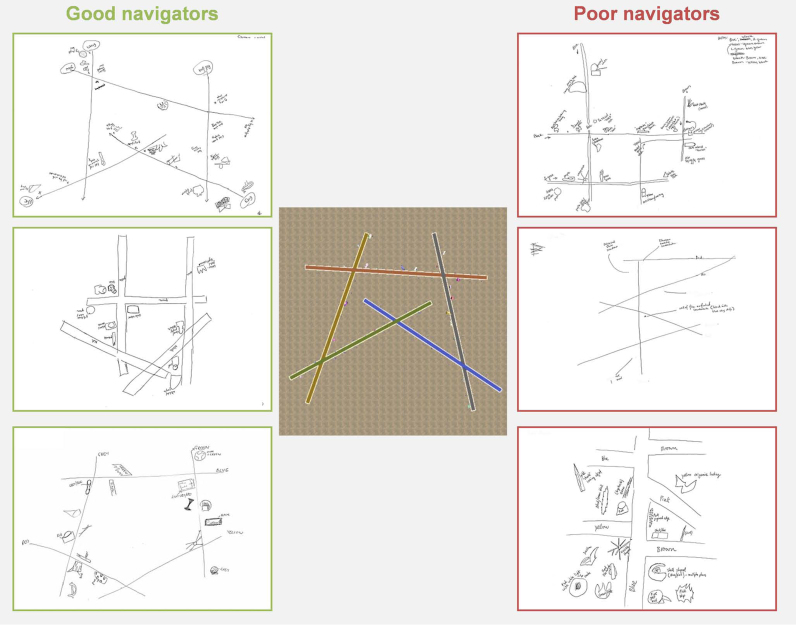
*Example sketch maps of good and poor navigators.* The actual layout of the environment is shown in the centre panel. On the left, edged in green, are example sketch maps drawn by good navigators. On the right, edged in red, are sketch maps drawn by poor navigators.

The post-scan navigation task provided the most direct way to examine navigation performance, and so a median split was used on this task's scores to determine the good and poor navigator groups. Full details of the two groups, their test scores and between-group comparisons (Bonferroni corrected) are provided in Table 1. While the groups were defined based upon performance in the navigation task, similar significant differences between the two groups were apparent in the sketch map test of the paths (where landmark knowledge was not required – see Fig. 2) and the landmark placement test. This is despite the two groups being very closely matched on age, sex, reasoning ability, visual memory and video game playing experience. Moreover, their self-reported navigation ability and ratings of landmark size, landmark visual salience and task difficulty were also comparable.

**Table 1:** Characteristics and performance data of the good and poor navigators.

**Measure**	**Good navigators Mean (SD)**	**Poor navigators Mean (SD)**	**t**_**(30)**_	**p value**
***General Characteristics***				
**n**	16	16	–	–
**Sex**	8 females	8 females	–	–
**Age**	23.8 (2.6)	23.6 (2.2)	0.147	0.9
**Visual memory**^a^	24.2 (4.9)	23.6 (5.0)	0.358	0.7
**Abstract reasoning ability (scaled score)**^b^	13.8 (1.2)	12.8 (1.9)	1.895	0.07
**SBSOD score**^c^	5.11 (0.9)	4.74 (1.2)	0.968	0.3
***Debrief Data***				
**Landmarks recognised (% correct)**	95.0 (4.8)	91.1 (11.4)	1.245	0.2
**Landmark permanence (% correct)**	73.5 (10.9)	57.5 (10.6)	4.235	**0.0002**
**Landmark size (1/small…3/large)**	2.1 (0.1)	2.1 (0.2)	0.113	0.9
**Landmark salience / attention-grabbing (1/not at all…3/a lot)**	2.1 (0.2)	2.0 (0.2)	0.404	0.7
**Sketch map task (max = 10, median = 5)**	6.5 (3.1)	3.25 (2.8)	3.129	**0.004**
**Landmark placement task (max = 75, median = 33.5)**	42.4 (10.6)	27.0 ( 5.9)	5.113	**<0.0001**
**Navigation task (max = 12, median = 3.5)**	5.9 (2.3)	1.6 (1.1)	6.691	**<0.0001**
***Debrief Ratings***				
**How difficult they found the task overall (1/very easy…5/very hard)**	4.2 (0.5)	4.4 (0.6)	−1.202	0.2
**How difficult they found learning the environment (1/very easy…5/very hard)**	4.2 (0.7)	4.4 (0.7)	−0.771	0.4
**Previous video game experience (1/none…5/very experienced)**	2.4 (0.9)	2.8 (1.3)	−0.963	0.3

Group means (and standard deviations) and between-group comparisons are shown. P values in bold denote significant group differences (Bonferroni corrected). Cohen's *d* effect sizes for significant results: landmark permanence = 1.5; sketch map task = 1.1; landmark placement task = 1.8; navigation task = 2.4.

a Visual memory was measured using the delayed recall of the Rey-Osterrieth Complex Figure (/36) (Osterrieth, 1944, Rey, 1941).

b Abstract reasoning ability was measured using the Matrix Reasoning sub-test of the Wechsler Abbreviated Scale of Intelligence (Wechsler, 1999).

c SBSOD = Santa Barbara Sense of Direction questionnaire (Hegarty et al., 2002).

### 3.2 Behavioural comparisons of landmark learning between good and poor navigators

Having divided the participants into good and poor navigators, and having also verified that there were no other major differences between the groups, we then went back to the fMRI phase to examine the subjects’ learning. There were 12 questioning periods during scanning (one associated with each learning ‘sweep’). We first divided them in to two (with 6 questioning periods in each), to reflect early and late learning phases. However, comparing the performance of good and poor permanence learners in the first half of learning, good learners were already significantly better at identifying the permanence of landmarks (1st half of learning: good mean (% correct)= 37.9, SD=6.6; poor mean=32.4, SD=7.8; t_30_=2.151; p=0.04; Cohen's *d*=0.8). We therefore thought it prudent to consider the data in finer detail, examining it in 3 learning segments (with 4 questioning periods in each) rather 2 for the following reasons: first, to check that there was no inherent difference in the performance of good and poor learners at baseline (i.e., that performance early on in learning was no different between the groups); second, to try and identify when the difference between good and poor learners first emerged.

**Fig. 3:**
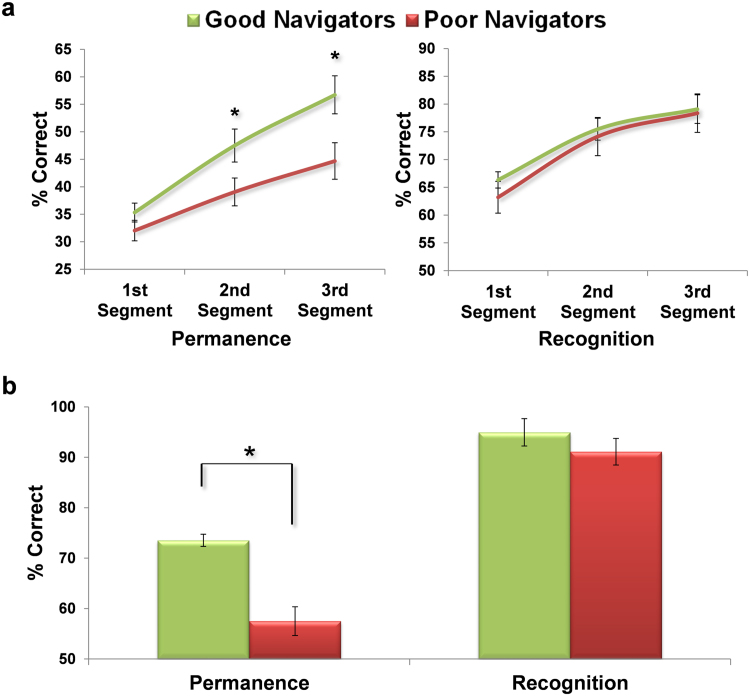
*Behavioural performance comparisons between good and poor navigators.* Good and poor navigator mean (+/− 1SEM) percentage correct responses during (a) the mini-tests in the fMRI scanner and (b) in the post-scan debriefing session, for permanence and recognition memory. Graphs on the left indicate knowledge of landmark permanence, and those on the right show landmark recognition performance. These data demonstrate that good and poor navigators did not differ in their ability to recognise landmarks, but poor navigators were significantly worse at registering their permanence (*p<0.05).

Examining the within-group learning of permanence, we confirmed that there was a significant effect of learning third on the registering of landmark permanence for both the good and poor navigator groups, (good: F_2,30_=30.776; p<0.0001; η_p_^2^=0.672; poor: F_2,30_=13.697; p<0.0001; η_p_^2^=0.477). However, comparing the two groups directly, we found there was a significant group (good/poor navigators) by learning segment (first/second/third) interaction (F_2,60_=3.911; p=0.03; η_p_^2^=0.088) (Fig. 3a, left panel). Post-hoc *t*-tests showed that while the two groups did not differ significantly in making this distinction in the first segment of the experiment (Permanence 1st segment of learning: good mean (% correct)=35.3, SD 6.9; poor mean=32, SD 7.5; t_30_=1.291, p=0.2), by the second segment of learning, good navigators were significantly better at identifying permanent landmarks, and this group difference persisted during the final segment of learning (Permanence 2nd segment: good mean=47.5, SD 12; poor mean=39.1, SD 10.1; t_30_=2.153, p=0.04, Cohen's *d*=0.8; Permanence 3rd segment: good mean=56.7, SD 13.8; poor mean=44.7, SD 13.3; t_30_=2.509, p=0.02, Cohen's *d*=0.9). Of note, there was also a significant correlation between scores on the in-scanner learning of permanence and post-scan navigation performance, which was present as early as the first segment (1st segment: r=0.42, p=0.02; 2nd segment: r=0.571, p=0.0006; 3rd segment: r=0.614, p=0.0002; Supplemental Fig. 1a).

We also assessed how well participants learned to recognise the identity of landmarks based on their performance in the questioning periods. There was no significant group (good/poor navigators) by learning segment (first/second/third) interaction (F_2,60_=0.284; p=0.8). Examination of the data shows that both good and poor navigators’ recognition of landmarks improved over the course of learning, with no difference in the learning rate between the two groups (Fig. 3a, right panel): recognition 1st segment of learning: good mean (% correct)=66.3, SD=5.9; poor mean=63.2, SD=11.4; t_30_=0.971, p=0.3; Recognition 2nd segment: good mean=75.5, SD=7.9; poor mean=74.2, SD=13.7; t_30_=0.334, p=0.7; Recognition 3rd segment: good mean=79.1, SD=10.3; poor mean=78.4, SD=13.8; t_30_=0.167, p=0.9. This shows that the poor navigators were specifically compromised at learning permanence, and this is unlikely to be explicable by inattentiveness, given the matched learning between the groups for landmark identity. There was also no significant correlation between scores on the in-scanner learning of landmark recognition and post-scan navigation performance (1st segment: r=0.21, p=0.2; 2nd segment: r=0.242, p=0.2; 3rd segment: r=0.253, p=0.2; Supplemental Fig. 1b).

Participants’ ability to recognise landmarks and identify if they were permanent or transient was tested again after the learning session in the scanner was over (Fig. 3b). At this point good (mean % correct=95, SD=4.8) and poor (mean % correct=91.1, SD=11.4) navigators were excellent at recognising landmarks from Fog World from among lure landmarks, with no difference between the groups (t_30_=1.25; p=0.2). However, good navigators were significantly better at identifying which landmarks were permanent (mean % correct=73.5, SD=10.9) compared to poor navigators (mean % correct=57.5, SD=10.6; t_30_=4.24, p=0.0002, Cohen's *d* = 1.5). As expected, correlations between post-scan landmark permanence and the three navigation-related post-scan measures were significant: vs Sketch Map: r = 0.523, p = 0.002; vs Landmark Placement: r = 0.785, p = 0.0001; vs Navigation Task: r = 0.784, p < 0.0001.

### 3.3 MRI scanning analyses

We first used VBM (Ashburner and Friston, 2000) to ascertain whether there were any structural brain differences between good and poor navigators either within our predefined ROIs (RSC, PHC, and HC) or anywhere else in the brain that might explain the landmark permanence finding, given previous associations of grey matter volume with navigation ability (Bohbot et al., 2007, Maguire et al., 2000, Schinazi et al., 2013, Woollett and Maguire, 2011). However, there were no group differences either within the ROIs or across the brain. This remained the case when a more liberal threshold (uncorrected p<0.001) was employed.

Considering next the fMRI data, similar to previous experiments (Auger et al., 2012, Auger and Maguire, 2013, Chadwick et al., 2015, Doeller et al., 2008, Janzen and van Turennout, 2004, Konkle and Oliva, 2012, Schinazi and Epstein, 2010, Wolbers and Buchel, 2005), we compared fMRI responses while subjects viewed images of individual, isolated landmarks displayed during the questioning periods at the end of each learning sweep. Using this time period, rather than when landmarks were viewed during the navigation movies, removed potential issues associated with visual confounds (e.g., path colour) and the more unconstrained neural responses that may have been associated with the minute-long learning movies. As with the behavioural learning analysis outlined previously, the fMRI learning data were divided into two halves (early learning: sweeps 1–6; later learning: learning sweeps 7–12).

**Fig. 4:**
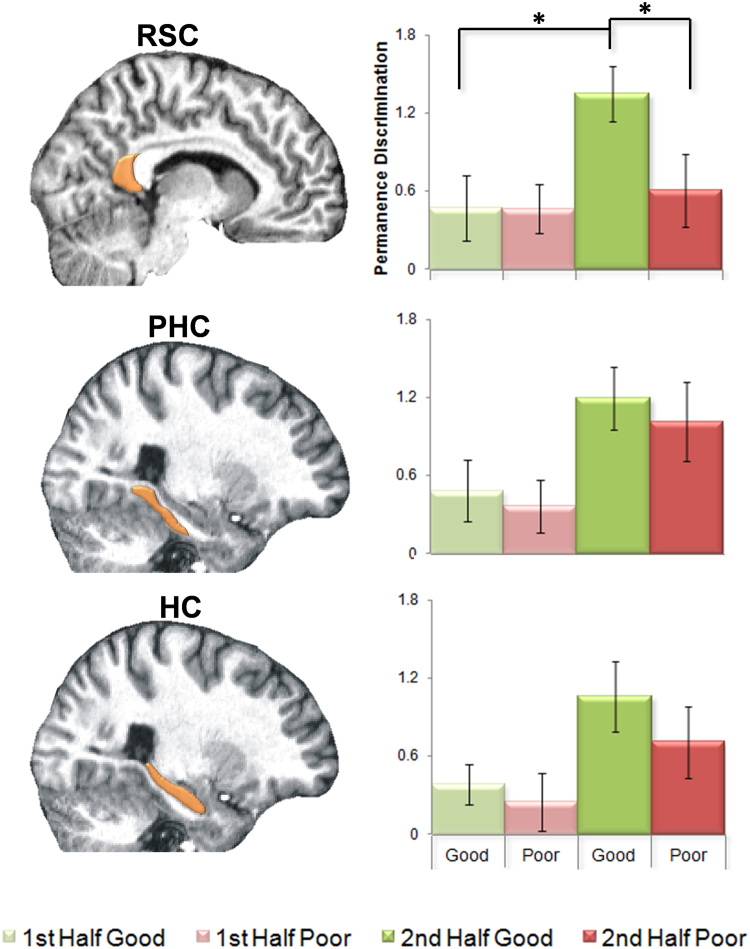
*fMRI comparisons between good and poor navigators.* Graphs show the mean (+/− 1SEM) difference in fMRI BOLD response (in arbitrary units) for permanent and transient landmarks in the retrosplenial cortex (RSC; top), parahippocampal cortex (PHC; middle) hippocampus (HC; bottom) and of good (green) and poor (red) navigators in the first (light shading) and second (dark shading) halves of learning. On the left, the locations of the three brain regions are indicated on a sagittal slice of a single representative subject's structural MRI scan. In the RSC, but no other region, good navigators showed a significantly greater difference in response between permanent and transient landmarks compared to poor navigators (*p<0.05).

Only in RSC was there a significant group (good/poor navigators) by learning segment (first half/second half) interaction, indicating a significant difference in how good and poor navigators’ responses to permanent and transient landmarks changed over the course of learning (RSC: F_1,30_=4.412, p=0.04, η_p_^2^=0.084; PHC: F_1,30_=0.489, p=0.5, η_p_^2^=0.001; HC: F_1,30_=0.685, p=0.4, η_p_^2^=0.006) (Fig. 4). Post-hoc *t*-tests revealed that in the first half of the learning phase, the responses of good and poor navigators did not differ in their permanence discrimination in any of the three regions: RSC: t_30_=0.018, p=0.99; PHC: t_30_=0.375, p=0.71; HC: t_30_=0.503, p=0.62. Therefore, it was not necessary to divide the fMRI data into three learning segments as in the behavioural data reported previously. However, the RSC of good navigators then went on to develop significantly greater discriminatory responses between the permanent and transient landmarks in the second half of learning compared with the first half (t_15_=2.507, p=0.02, Cohen's *d*=1.0). This did not happen in the RSC of poor navigators (t_15_=0.525, p=0.6), or in the PHC or HC of either good (PHC t_15_=1.677, p=0.1; HC t_15_=1.946, p=0.07) or poor (PHC t_15_=1.916, p=0.08; HC t_15_=1.319, p=0.2) navigators. Of note, we also divided the hippocampus into anterior and posterior portions but, as with the whole hippocampus, no difference between good and poor navigators emerged (Supplemental Fig. 2).

This meant that during the second half of the learning phase, RSC responses of good navigators discriminated the permanence of landmarks significantly more than that of poor navigators (t_30_=2.112, p=0.04, Cohen's *d*=0.7), but there were no such differences in either of the other regions (PHC: t_30_=0.473, p=0.6; HC: t_30_=0.908, p=0.4). Therefore, the less effective learning of landmark permanence by poor navigators was associated with decreased discriminatory responses for permanent and transient landmarks specifically within the RSC.

### 3.4 Mediation analysis

To further explore the relationship between permanence learning, navigation and fMRI activity within the three ROIs, we conducted mediation analyses. We specifically assessed whether fMRI activity in the ROIs might mediate a relationship between permanence learning and subsequent navigation performance. There was a significant effect of permanence learning on navigation through RSC activity (mediator effect 0.237, p=0.01). There was also a smaller significant mediator effect for HC activity (0.0183, p=0.01). PHC activity showed no significant mediator effect (−0.018, p=0.2). In other words, the RSC mediator effect could account for 24% of the total effect permanence learning had upon navigation, HC accounted for less than 2% and PHC contributed nothing.

## 4 Discussion

In this study participants had to learn a new virtual environment by repeatedly moving through it. Our interest was in good and poor navigators – those who were successful or not at finding their way within this environment after the learning phase. We found that those who were poor at wayfinding were impaired, compared to good navigators, at registering whether landmarks were permanent or transient during learning, and this was associated with reduced activity in RSC. This is despite memory for the landmarks and other background characteristics being similar between good and poor navigator groups. A mediation analysis formally demonstrated that there was a significant effect of landmark permanence learning on navigation performance mediated specifically through RSC activity. Thus, the impaired learning of landmark permanence seemed to significantly disadvantage the poor navigators compared to the good navigators, with the accuracy of their internal representation of the environment and their ability to navigate within it being compromised.

Previous reports of brain differences associated with navigation ability have been largely focussed upon the hippocampus (Bohbot et al., 2007, Hartley et al., 2003, Iaria et al., 2008, Janzen et al., 2008, Maguire et al., 2000, Schinazi et al., 2013, Woollett and Maguire, 2011). Our current results add to the growing realisation that the RSC plays a key role in navigation variability in concert with areas such as the hippocampus (Auger et al., 2015, Iaria et al., 2007, Maguire, 2001, Sulpizio et al., 2016, Vann et al., 2009, Wolbers and Buchel, 2005). Moreover, our data go further by characterising the nature of its contribution as possibly being centred upon the learning of landmark permanence, thus showing why the RSC may provide a vital foundation for spatial representations that enable efficacious navigation.

It has been shown previously that poor navigators are less consistent than good navigators at identifying well-known everyday outdoor items that are permanent and do not move within an environment (Auger et al., 2012, Auger and Maguire, 2013). While this suggested a relationship between the processing of landmark permanence and navigation ability, more direct evidence was lacking. Our results now show that people who go on to be poor at finding their way within an environment had difficulty learning landmark permanence during the initial building of a mental representation of that environment. Of note, this result cannot be due to poor navigators having deficient prior knowledge about permanent landmarks, because the landmarks we used here were entirely novel and participants could have no knowledge or expectations about their permanence. But perhaps other differences between the good and poor navigators can explain our findings.

As detailed on Table 1, another novel aspect of this study was how well matched the good and poor navigators were on a range of general intellectual and memory measures, as well as in terms of the number of males and females. On one measure of abstract reasoning ability, the good navigators, on average, scored higher than the poor navigators. However, this difference was not statistically significant, and both groups scored well above average on this task. The subjective perception of landmark features such as size and visual salience was similar for both groups, as was the perceived difficulty of the tasks. Perhaps poor navigators merely paid less attention or had general issues with learning during the fMRI scan. If this was the case then we might have expected them to be worse, or slower, than good navigators at learning to identify landmarks from among lures. However, the two groups performed very well on this task during and after the learning phase, with no differences between good and poor navigators. Structural brain differences were also not evident between the groups. The only distinction between the groups was in the learning of landmark permanence, and this correlated with their subsequent navigation performance. This provides novel evidence concerning what particular component of landmark learning may influence subsequent individual differences in navigation performance.

We considered three brain regions of interest, motivated by previous studies that have linked them to navigation ability - RSC, PHC and HC (Auger et al., 2012, Auger and Maguire, 2013, Bohbot et al., 2007, Iaria et al., 2008, Janzen et al., 2008, Maguire et al., 2000, Schinazi et al., 2013, Woollett and Maguire, 2011). In the first half of learning there were no differences in activity in any of the regions between the good and poor navigator groups in response to landmark permanence. In the second half of learning, there was generally increased activity in all of the regions, but only the RSC differentiated the good from the poor navigators. The mediation analysis adds further weight to this finding by showing that there was a significant effect of landmark permanence learning on navigation performance mediated specifically through RSC activity. HC activity had a much smaller mediation effect and PHC activity had none. Overall, therefore, our results present the strongest evidence yet of the RSC supporting the processing of landmark permanence that then seems to have an effect on the efficacy of navigation.

If the RSC-mediated process of distinguishing permanent from transient landmarks is poor, then areas like the hippocampus may not be able to perform their functions effectively either, thus leading to generalised spatial disorientation. Our findings suggest that environmental representations of poor navigators might be built upon inappropriate, non-permanent landmarks, constraining the efficacy of the navigation process at an early stage. But it could be argued that our poor navigators did not have an issue with permanence per se, but rather with encoding of the specific spatial locations that the permanent landmarks were associated with. We feel this is unlikely for two reasons. First, previous studies that examined landmark features, presented everyday outdoor items in isolation, with no background or location associated with them. Nevertheless, poor navigators were less reliable than good navigators at identifying specifically landmark permanence and this was associated with reduced RSC engagement (Auger et al., 2012, Auger and Maguire, 2013). Second, a previous study showed that landmark permanence-related responses developed initially in RSC, after which increased coupling was noted between RSC and HC, with HC, but not RSC, then expressing knowledge of permanent landmark locations (Auger et al., 2015).

If the RSC is not responding to landmark locations, then how should we conceptualise its response to permanence? The neural mechanisms underpinning the RSC's response to landmarks and its electrophysiological properties, particularly in primates, remain under-explored (Aggleton, 2010, Buckley and Mitchell, 2016, Vann et al., 2009). It could be linked to the presence of head direction cells which have been reported within the rodent RSC (Chen et al., 1994, Cho and Sharp, 2001, Jacob et al., 2017), with similar representations also reported in humans (Marchette et al., 2014, Shine et al., 2016). This suggests that head direction cell firing may be centred upon reliable predicable features such as permanent landmarks, and this information is integrated within the RSC (Auger et al., 2012, Auger and Maguire, 2013, Jeffery et al., 2016).

Two other features of this study should also be considered in interpreting our results. The first is the way in which we divided the participants into two groups. Our central interest was in good and poor navigators, so those who were either good or bad at wayfinding. One way to identify such individuals is to have them subjectively rate their own navigation ability using an instrument such as the Santa Barbara Sense of Direction (SBSOD) questionnaire. Unlike our previous experiments where the SBSOD questionnaire was administered after the experiments were concluded (Auger et al., 2012, Auger and Maguire, 2013), in the current study it was administered prior to the experiment commencing. The good navigator group, on average, had a higher score on the SBSOD than poor navigators, but the difference was not statistically significant. This accords with the findings of Heth et al. (2002) who demonstrated that pre-test self-reporting of navigation ability has little relationship with actual navigation ability compared to when self-reporting occurs after testing.

The most obvious and objective way to identify such individuals was to have them learn an environment and then test their ability to navigate to locations within it. This has close parallels with how people behave in the real-world, and encompasses many aspects of an environmental knowledge including paths, their relationships, landmark types and their locations. We therefore used performance on the post-scan navigation test to identify good and poor navigators. It could be argued, however, that this test was biased towards knowledge of landmark locations, but then so is real-world navigation. However, the sketch map test was scored solely on the basis of the relationships of the paths that comprised Fog World, ignoring landmarks. Nevertheless, on this task too, the performance of the poor navigators was significantly worse than that of the good navigators, who showed a much better appreciation of the environment's overall layout. Hence we believe our method of identifying the two groups was appropriate and pragmatic.

The second feature to consider about our study is the nature of the virtual environment. We deliberately used a sparse environment which allowed us to precisely control the exposure to every path, junction and landmark, and which minimised other extraneous factors. Of course this environment is consequently less naturalistic, but for the first study of its kind examining the link between learning of landmark permanence in good and poor navigators, we felt it was necessary and logical to adopt a controlled approach in this instance. In the future it will be important to examine whether similar results pertain in more naturalistic virtual environments, where it might also be possible to look at the effects of individual exploration strategies (Etchamendy and Bohbot, 2007, Spiers and Barry, 2015). Future work in more naturalistic settings could also consider the interactions between multiple relevant factors, in addition to landmark permanence, which may impact upon the efficacy of navigation, such as the ability to recognise landmarks located at navigationally relevant locations (Janzen et al., 2008) and path integration (Chrastil et al., 2017).

It is interesting to note that the preservation of the poor navigators’ ability to recognise landmarks mirrors what is observed in people with lesions involving the RSC (Maguire, 2001, Vann et al., 2009). These patients have “topographical disorientation”, whereby they are unable to derive navigational information from landmarks while showing no apparent impairment at recognising them. Similarly, those with a congenital “developmental topographic disorientation” have a lifelong navigational impairment associated with altered RSC function (Kim et al., 2015). This also has relevance for patients with Alzheimer's dementia, where some of the earliest pathological brain changes are centred upon the RSC (Nestor et al., 2003, Pengas et al., 2010, Tan et al., 2013, Tu et al., 2015), and spatial disorientation is a common initial symptom. The deficits in navigation experienced by these various populations with RSC pathology could perhaps be underpinned by aberrant processing of landmark permanence.

Our results do not inform about whether the poor navigators were impaired at registering permanent landmarks because their RSC was fundamentally limited in how much it could activate, or whether the RSC simply did not engage to its full potential because the poor navigators did not register stable landmarks for cognitive-strategic reasons. However, identifying this link between wayfinding ability and processing of landmark permanence opens up potentially new opportunities to try and improve the efficacy of navigation. If poor navigation ability is at least partly explained by a failure to form reliable representations of environments by basing them upon inappropriate, non-permanent landmarks, it might be possible to ‘train’ poor navigators to learn to use permanent environmental features. This kind of intervention might even be beneficial for spatial orientation in the context of Alzheimer's dementia, at least in the early phase of the disease.

In conclusion, despite broad acceptance that environmental representations are built upon permanent, stable landmarks (Burnett et al., 2001, Lynch, 1960, Siegel and White, 1975), variability in the processing of permanent landmarks as a potential source of individual differences in navigation has been relatively neglected. Further work investigating this link, and the role played by the RSC, could illuminate how spatial representations are built, how they vary and whether they are amenable to change.

## Author contributions

All authors were involved in the conception and design of the experiment, as well as analysis and interpretation of the data. S.D.A. acquired the data. The manuscript was written by S.D.A. and E.A.M. and was reviewed by all the authors.

## Competing financial interests statement

The authors declare no competing financial interests.
