# Supplementary material for: Efficacy of navigation may be influenced by retrosplenial cortex-mediated learning of landmark stability
Source: Neuropsychologia. 2017 Sep;104:102–12. doi: 10.1016/j.neuropsychologia.2017.08.012 (PMC5637158; doi:10.1016/j.neuropsychologia.2017.08.012)
Supplement: Supplementary file 1 — Supplementary material [file mmc1.pdf]

Auger et al. SUPPLEMENTAL FIGURES

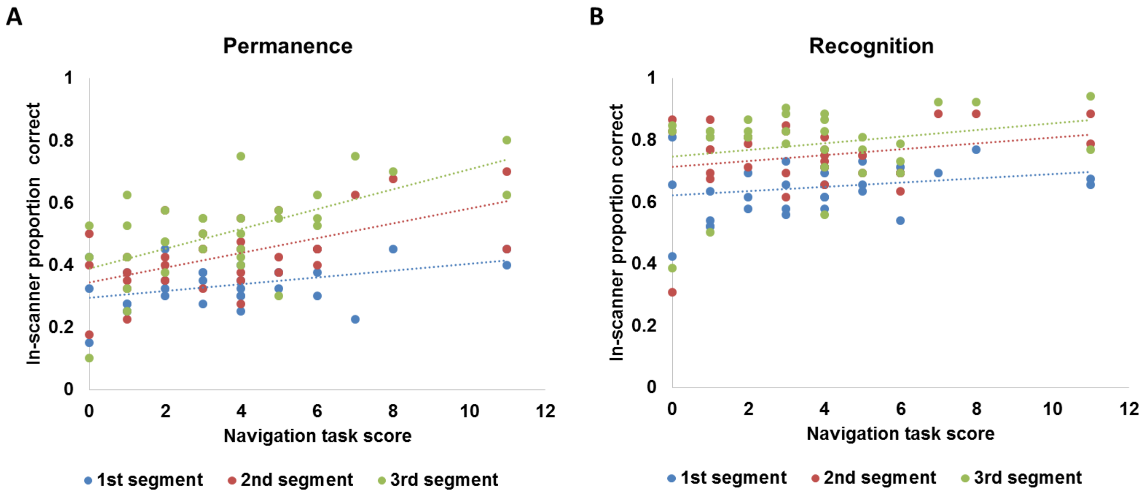

Supplemental Figure 1. Scatter plots demonstrating the correlation between post-scan navigation performance and scores on the in-scanner learning of landmark permanence (A) and recognition (B) in each of the three learning segments. For permanence, all correlations were significant (1st segment: r=0.42, p=0.02; 2nd segment: r=0.571, p=0.0006; 3rd segment: r=0.614, p=0.0002). For recognition, none were significant (1st segment: r=0.21, p=0.2; 2nd segment: r=0.242, p=0.2; 3rd segment: r=0.253, p=0.2).

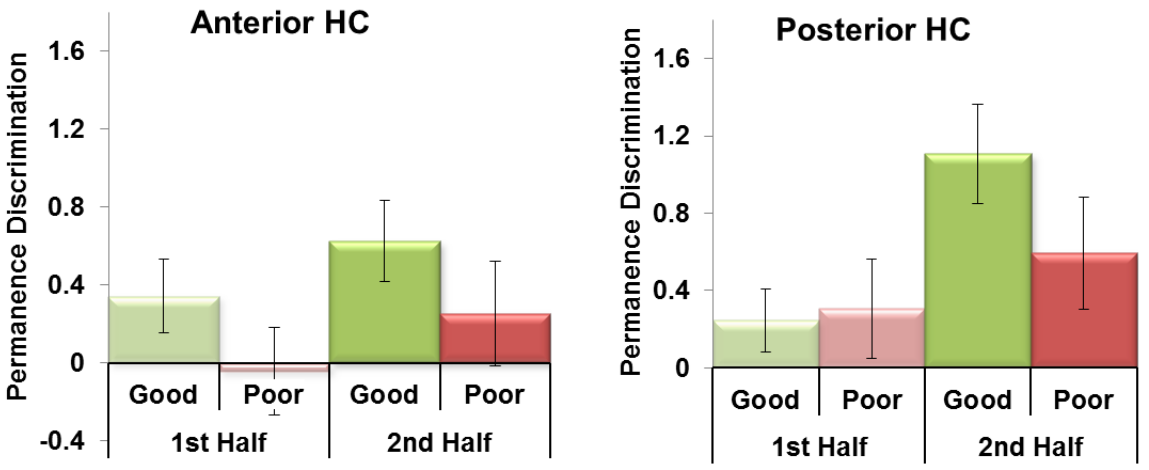

Supplemental Figure 2. Graphs show the mean (+/- 1SEM) difference in fMRI BOLD response (in arbitrary units) for permanent and transient landmarks in anterior HC (left) and posterior HC (right), of good (green) and poor (red) navigators in the first (light shading) and second (dark shading) halves of learning. The graphs suggest that responses in the posterior HC mirror the results from the whole HC more closely than the anterior HC. While this may be consistent with other work implicating the posterior hippocampus in particular in spatial processing (e.g., Maguire et al., 2000), there were no significant differences between good and poor navigators.

We also ran separate mediation analyses for the anterior and posterior hippocampus. Both showed small but significant mediator effects (anterior: 0.0134, p=0.02; posterior: 0.0162, p=0.03). The posterior hippocampus had a slightly larger mediator effect than anterior, but this was less than the hippocampus as a whole (where the mediator effect was 0.0183, p=0.01).

Maguire, E.A., Gadian, D.G., Johnsrude, I.S., Good, C.D., Ashburner, J., Frackowiak, R.S.J., Frith, C.D., 2000. Navigation-related structural change in the hippocampi of taxi drivers. Proc. Natl. Acad. Sci. U. S. A. 97, 4398–4403.
